# Frozen Zoo: a collection of permafrost samples containing viable protists and their viruses

**DOI:** 10.3897/BDJ.8.e51586

**Published:** 2020-07-10

**Authors:** Stas Malavin, Lyubov Shmakova, Jean-Michel Claverie, Elizaveta Rivkina

**Affiliations:** 1 Soil Cryology Lab, Institute of Physicochemical and Biological Problems in Soil Science RAS, Pushchino, Russia Soil Cryology Lab, Institute of Physicochemical and Biological Problems in Soil Science RAS Pushchino Russia; 2 Aix-Marseille University, CNRS, IGS (UMR7256), IMM (FR3479), Marseille, France Aix-Marseille University, CNRS, IGS (UMR7256), IMM (FR3479) Marseille France

**Keywords:** Permafrost, microbiology collection, protists, giant viruses

## Abstract

**Background:**

Permafrost, frozen ground cemented with ice, occupies about a quarter of the Earth’s hard surface and reaches up to 1000 metres depth. Due to constant subzero temperatures, permafrost represents a unique record of past epochs, whenever it comes to accumulated methane, oxygen isotope ratio or stored mummies of animals. Permafrost is also a unique environment where cryptobiotic stages of different microorganisms are trapped and stored alive for up to hundreds of thousands of years. Several protist strains and two giant protist viruses isolated from permafrost cores have been already described.

**New information:**

In this paper, we describe a collection of 35 amoeboid protist strains isolated from the samples of Holocene and Pleistocene permanently frozen sediments. These samples are stored at −18°C in the Soil Cryology Lab, Pushchino, Russia and may be used for further studies and isolation attempts. The collection strains are maintained in liquid media and may be available upon request. The paper also presents a dataset which consists of a table describing the samples and their properties (termed "Sampling events") and a table describing the isolated strains (termed "Occurrences"). The dataset is publicly available through the GBIF portal.

## Introduction

Permafrost, or perennially frozen ground, is the ground that remains below zero degrees Celsius for two or more consecutive years (Goudie 2004, p. 777). In North-Eastern Siberia, the age of the permafrost may reach millions of years and span 1 km below the surface of the ground (Gilichinsky and Rivkina 2011). In this ancient permafrost, viable prokaryotic and eukaryotic organisms have been found (Rivkina et al. 2007, Gilichinsky et al. 2008, Ozerskaya et al. 2009, Shatilovich et al. 2009, Vishnivetskaya 2009, Jansson and Taş 2014, Shatilovich et al. 2015, Shmakova and Rivkina 2015, Shcherbakova et al. 2016). In the case of syncryogenetic formation, i.e. simultaneous sedimentation and freezing, the age of deposition of all particles in a layer is approximately the same (Gilichinsky and Rivkina 2011). Thus, by observing sterile conditions during all stages of sampling and cultivation, it is possible to date isolated microorganisms by the age of the sediments.

The sediments, from which living microorganisms have been successfully isolated, date back to a million years BP. These strains are of great scientific interest for several reasons. First, this is a remarkable case of the organism's hardiness, far exceeding the traditional view on how long an organism can survive, even in the suspended stage of the life cycle. Although subzero temperatures down to −50°C are not incompatible with certain metabolic reactions in bacteria (Rivkina et al. 2000, Bakermans et al. 2003, Price and Sowers 2004, Johnson et al. 2007, Rohde et al. 2008), including even DNA replication (Tuorto et al. 2014), the main factor limiting the metabolism in permafrost deposits is the lack of sufficient amount of liquid water (Rivkina et al. 2000). Concerning the spore-forming bacteria and cyst-forming protists (to which 100% of protists reported from permafrost belong), those are likely to remain in these deposits in a state of cryptobiosis, or “hidden life”, which involves certain biochemical adaptations to endure adverse factors. Second, as cysts do not replicate, protists trapped in the permafrost are therefore excluded from the evolutionary process. Thus, the comparison of closely related strains isolated from sediments of different age and modern soils may allow the study of evolutionary changes that have happened since the time of sedimentation. Finally, strains from permafrost are the remnants of the ancient ecosystems that have disappeared. During the thawing of permafrost on outcrops along the banks of rivers and seas, organisms from ancient sediments are likely to penetrate into modern ecosystems with not totally understood consequences (Gubin et al. 2003, Graham et al. 2012).

Amoeboid protists, a polyphyletic group of eukaryotic, mostly unicellular, microorganisms with inconstant cell shape, are an important component of all soil ecosystems (e.g. Geisen et al. 2018). They primarily feed on bacteria and other protists, but can also consume dissolved organic substances (Stockem and Klein 1979). Amoeboid protists are absolutely ubiquitous, with many genera characterised by worldwide distribution (e.g. Smith and Wilkinson 2007). To date, we have isolated about 40 strains of amoeboid protists, mostly from Amoebozoa and Heterolobosea, from permafrost samples. We have also encountered amoeboflagellates from the Cercozoa supergroup, but have not isolated them due to time and resources limits. Shatilovich et al. (2010) also reported cercozoans from nesting chambers of the ground squirrel (*Urocitellus* sp.) burrows found frozen in permafrost. In our isolation experiments, only about 5% of samples yielded live strains (compared to the usual 100% of modern soil samples), so such isolation constitutes a relatively rare event. The samples, from which the strains have been isolated, remain frozen and further isolation from them is possible, as shown by our experiments. Additionally, this makes possible some direct environmental measurements—a property rarely represented in microbiological collections—as well as the analysis of total DNA, either by metabarcoding or metagenomics. Some data on prokaryotic microorganisms obtained by shotgun metagenomics from permafrost samples are published by Rivkina et al. (2016).

The data on the taxonomic composition of amoeboid protists in the Arctic and Antarctic are scarce compared to the better-studied temperate areas. Brown et al. (1982) have found amoebae from the genera *Acanthamoeba*, *Hartmanella*, *Platyamoeba*, *Naegleria* and *Vahlkampfia* in the Antarctic. Smith (1982) discovered *Phalansterium*, *Mayorella*, *Metachaos*, *Vanella*, *Vexillifera*, *Tetramitus*, *Naegleria*, *Vahlkampfia* and 10 genera of testate amoebae in the Sub-Antarctic island of South-Georgia. Notably, a first solitary species of *Phalansterium*, *P. solitarium*, was described from Svalbard (Sandon 1924). Several strains of heterolobose amoebae from both the Antarctic and Arctic soils have been described since then (De Jonckheere 2006, Robinson et al. 2007, Tyml et al. 2016). However, more attention has been paid to the testate taxa due to their role as markers in bioindication and paleoenvironmental reconstruction (Beyens et al. 1986, Vincke et al. 2004, Tsyganov et al. 2011, Taylor et al. 2019). Antarctic fauna have been studied better than the fauna of the Arctic, with even a checklist published recently (Thompson et al. 2019). Concerning Northern Siberia, the available data are almost exclusively limited to testate amoebae (Beyens and Chardez 1995, Smith et al. 2007, Bobrov and Wetterich 2012, Shmakova et al. 2013). Our collection therefore contributes to the study of Arctic fauna of amoeboid protists.

The finding of live protists in the permafrost layers up to hundreds of thousands years old significantly expands our view on the survival capabilities of eukaryotes and raises questions about the mechanisms that make this survival possible. Despite intensive research during the last 40 years, our understanding of these mechanisms in unicellular organisms is still far from completion Bernhard et al. 2010, Anderson 2016, Souffreau et al. 2019. Permafrost presents us with the results of a monumental experiment impossible to set up in the laboratory; and the collection we describe allows us to study these results in any detail. Moreover, strains isolated from permafrost constitute the remains of a disappeared ecosystem. This, first, allows us to study the taxonomic and functional diversity of that ecosystem and, second, to compare the isolated "ancient" strains with their modern relatives at any desired level to study the evolution Shmakova et al. 2016.

An interesting peculiarity about the described samples is the isolation of two new giant double-stranded DNA viruses from one of them (Legendre et al. 2014, Legendre et al. 2015). Giant viruses were so-called because they are visible by light microscopy, but they also have the largest genome sizes and gene contents known amongst viruses. Amongst those genes are transfer RNA genes, translation initiation factor genes and other remnants of translation machinery that are not recent transfers from cellular organisms. The giant viruses isolated from permafrost samples, *Mollivirus sibericum* and *Pithovirus sibericum*, presumably represent new giant virus families (Legendre et al. 2014, Legendre et al. 2015).

## Sampling methods

### Study extent

The samples were collected in the field and stored constantly frozen during all periods of transportation and processing. In the lab, a part of each sample was used to isolate protist strains. The remaining part has never been melted and is stored at −18°C. The isolation was done in sterile conditions. Revived protists were cloned and are maintained as bacterised or axenic cultures on plastic or agar with liquid overlay.

### Sampling description

**Figure 2.**
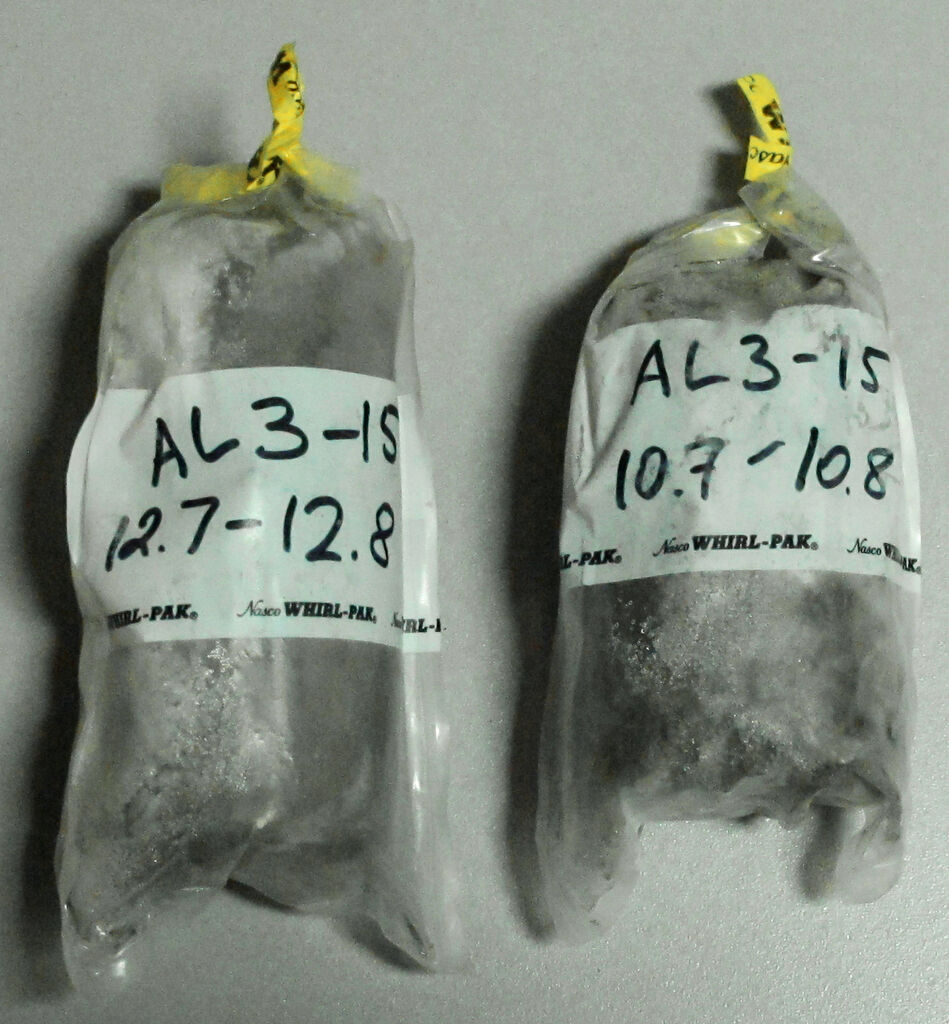
Core samples in sterile bags.

Drilling was performed using a mobile drilling rig (core-drilling machine) UKB-12/25 (V.V. Vorovsky Machine-Building Plant, Moscow, Russia) operated without flushing and blowing (Fig. 1a). Flushing and blowing were shown to cause contamination of the cores by modern soil microorganisms (Gilichinskiy et al. 1989). Each core was collected every 30–70 cm of the drilling. The core diameters were 115 to 75 mm, depending on the well depth (the deeper the well, the smaller the core). Removed cores were wrapped in a one-centimetre-thick coat of half-melted cuttings. Immediately after collection, this coat was removed with a knife, showing a completely frozen inner part. After a short lithological and glaciological description of the sediments, each core was passed to a clean field lab organised in a tent. Operations in the lab were conducted behind a gas-fired burner using disposable materials and gloves, following general microbiological practice. In the lab, the core was shaved with a sterile scalpel so that a 5 mm outer layer was removed. The remaining core was 4-6 cm in diameter, depending on the initial value. Immediately after shaving, the core was placed into a sterile sampling bag (Fig. 2) and placed in a portable freezer, a cave dug into an ice wedge or an empty borehole used as a freezer. In total, the “outdoor” stage of the process lasted no more than 10 minutes, depending on the current well depth. The “indoor” lab stage took around 5 minutes. All collected cores were kept at negative temperatures during the whole period of transportation to the stationary lab.

Outcrops are natural exposures of permafrost sediments formed at sea and river banks. The advantage of sampling from the outcrop wall is the possibility of visual inspection and description of the whole sediment layer, including preserved soils (Fig. 3a). Samples from outcrops were taken from the frozen surface of the outcrop wall after the removal of melted material. In the wall, a hole of about 40 cm deep was made with either a hand-held drill (Fig. 1b), a chisel or a knife. A cylinder sample about 5 cm in diameter was carefully carved or drilled out from the bottom of the hole, treated with 95% ethanol and immediately placed into a sterile plastic bag (Fig. 2). As with the cores, all collected outcrop samples were kept frozen during the whole period of transportation.

Buried terminal nesting chambers of ground squirrel (*Urocitellus* sp.) burrows (Fig. 3b) are unique paleontological objects of Pleistocene Ice Complex sediments. They usually contain animal supplies made of seeds of surrounding grasses. Usually frozen in the living state, they are very well preserved. From the tissue of a *Silene* sp. seed found in a buried nesting chamber, a viable flowering plant was grown (Yashina et al. 2012). Nesting chambers also contain a diverse community of protists and fungi. Chambers were cut from the outcrop wall in one or several pieces, each 10 or more cm in dimension, put immediately in sterile plastic bags and kept frozen until processing in the lab.

### Quality control

During the development of the permafrost microbiological sampling technique, several tests for contamination of the core interior were established at different phases of sampling and storage. Gilichinskiy et al. (1989) and Shi et al. (1997) used a bacterium, *Serratia marcescens*, which produces easily noticeable red colonies. The drilling barrel was covered with a culture suspension 2 h prior to drilling. In a parallel experiment, frozen samples were seeded with the same suspension in the lab and left intact at negative temperature for several hours to several months. The distribution of *S. marcescens* cells in a core was investigated during sample processing. In both tests, bacteria have been found exclusively in the outer layer and never inside the core.

Later, Juck et al. (2005) used fluorescent latex beads (microspheres), 0.5 μm in diameter and a transformed *Pseudomonas* strain expressing green fluorescent protein (GFP). Both beads and transformed bacteria were applied to the drilling equipment before drilling, similarly as described above for *S. marcescens* suspension. Fluorescence microscopy showed that neither beads nor bacteria penetrate a sample more than 1 cm below the surface. Additionally, polymerase chain reaction revealed no amplification of the GFP gene from the inner part of the cores.

Based on the negative results obtained for bacteria and fluorescent beads, i.e. particles around 2 μm in diameter or less, we consider that protist cysts, which are at least five times larger, cannot move inside the frozen ground and thus could not have penetrated the sediments much later than they were deposited. In the same way, the contamination of the inner part of the samples during sampling and laboratory processing is highly unlikely.

## Geographic coverage

### Description

**Figure 4.**
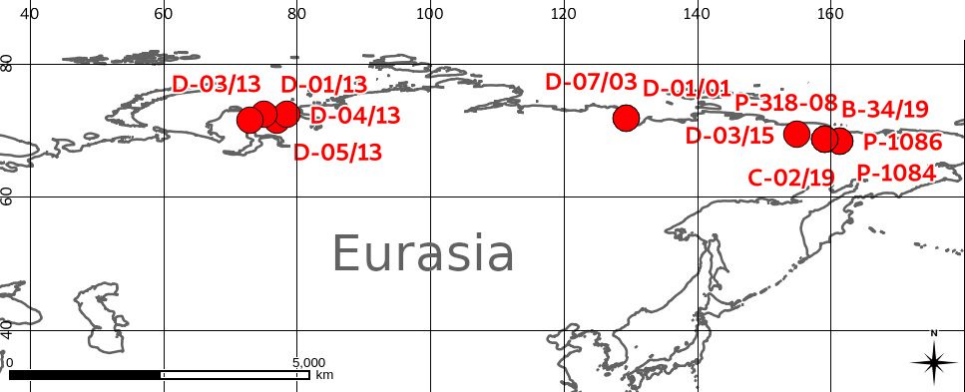
Location of sampling events. North-Eastern Eurasia.

The samples were obtained from three areas in High Eurasian Arctic, i.e. the Gydan Peninsula, the Bykovskiy Peninsula and the Kolyma Lowland (Fig. 4). Locations of the sampling sites are present in Table 1 (entries in the "Parent event id" column correspond to the labels in Fig. 4).

## Taxonomic coverage

### Description

We isolated protists from permafrost samples using enrichment cultivation. Specifically, three portions of ca. 1 cm^3^ from the inner part of each frozen sample were placed into 90-mm Petri dishes filled with 10 ml autoclaved mineral Prescott and James (PJ) medium (Prescott and James 1955). Negative controls, i.e. same procedures without sample inoculate, were set up simultaneously. The isolation was performed in a laminar flow hood using disposable or sterilised equipment. After incubation of one week, samples were examined using a Nikon TS-100 inverted microscope. Detected cells were cloned by transferring them individually to a new dish using a disposable glass capillary and subsequently re-cloned several times. The resulting strains were cultured in 60 mm Petri dishes using modified 0.1% Cerophyl infusion made on PJ medium (Smirnov and Brown 2004).

Preliminarily, we identified isolated strains to the lowest possible level using keys and diagrams as in Smirnov and Brown 2004, Patterson 2003, Page 1988. Branching and network-forming amoebae (BNFA) were not identified to any level. Further study of certain strains involved electron microscopy and molecular phylogeny (referenced in Table 2). In total, we isolated 34 strains belonging to Amoebozoa and Heterolobosea and one testate strain belonging to Cercozoa. Cercozoan amoeboflagellates were observed in enrichment cultures, but were not isolated due to time and resource limits. Higher taxonomy (following Adl et al. 2018), source of identification, location of sampling and the estimated age of the isolated protist strains and viruses are presented in Table 2. Age estimation is detailed below in the "Age of the isolated strains" section.

For the isolation of viruses, 400 mg of the sample were resuspended in 6 ml of PJ. Each 3 ml were supplemented with 300 μl of Amphotericin B (Fungizone) at 250 μg/ml. A volume of 1.65 ml of this solution was left overnight under stirring at 4°C. After decantation, the supernatant was centrifuged at 800×g for 5 min. *Acanthamoeba castellanii* strain Neff (ATCC 30010™) culture adapted to Fungizone was inoculated with 100 μl supernatant and with the pellet resuspended in 50 μ buffer (Tris 20 mM, CaCl 21 mM, pH 7.4). *Acanthamoeba* cells were cultured at 32°C in microplates with 1 ml of PPYG medium (Neff et al. 1964) supplemented with 100 μg/ml of ampicillin, 100 μg/ml of penicillin-streptomycin and 2.5 μg/ml of Fungizone and monitored for cell lysis. Virion factories inside host cells were visualised using TEM. Infection trials were performed twice and produced identical results (Legendre et al. 2014, Legendre et al. 2015).

When the cell lysis was completed, cultures were centrifuged for 5 min at 500×g to remove cellular debris and virus particles were pelleted by a 30-min centrifugation at 3,000×g. The pellet was then washed twice in PBS and centrifuged at 5,000×g for 15 min on a discontinuous sucrose gradient (30%/40%/50%/60% wt/vol). Purified particles were studied by scanning electron microscopy. Genomic DNA was recovered from 1.8 × 10^10^ purified particles and sequenced in paired-end flow cell on the Illumina MiSeq system using 151 base read chemistry. Viruses were identified and described based on their genome sequences, SEM of the virion morphology and TEM of the virion factory morphology (Legendre et al. 2014, Legendre et al. 2015).

## Traits coverage

**Age of the isolated strains.** The specific property of the described collection is that its strains are of ancient, mostly Pleistocene, origin and represent a part of a disappeared ecosystem. Due to the small number of cells in the frozen sediments, it is not possible to date these cells directly, thus an indirect method is needed. In the case of syncryogenetic formation, when freezing of the sediments occurs together with their deposition, all particles, including bacterial and fungal spores and protist cysts, become frozen at approximately the same time. If frozen deposits have not melted, which may be inferred from cryotexture, distribution of methane or other signatures, no particles of bacterial size or larger could have penetrated from the surface. Thus, one could assume the age of the cells trapped in permafrost to be roughly the same as the age of the permafrost itself or, in other words, that the found cells originate from the time of the last sediment freezing. This time may be determined by radiocarbon (^14^C) dating of carbon-containing remnants and substances produced by the biota before sedimentation-freezing occurred.

**Gydan Peninsula.** Based on radiocarbon and optically-stimulated luminescence (OSL) dating, sediments associated with massive ice formations in the Gydan Peninsula are considered to be of the Late Pleistocene estuarine-alluvial origin (Mahaney et al. 1995). However, Holocene formations are also present (Demidov et al. 2016). For instance, radiocarbon dating of the sample D-01/13-2.0 from 2 m depth of the borehole D-01/13 gave 8580 ± 50 years BP (Demidov et al. 2016). From the same borehole, samples collected at 6 and 10 m gave, correspondingly, 17000 ± 55 and 15000 ± 50 years BP. Such reversion is usually encountered in geochronological studies. It may be attributed to either the vertical transfer of substance, which in the case of syncryogenetic sedimentation does not occur, or to the different proportion of carbon sources, which differ in age considerably, sometimes by several thousand years (Brock et al. 2010). Thus, we consider the sediments of the sample D-01/13-8.0 (8 m) to be 15 to 17 thousand years old (Late Pleistocene). Sample D-01/13-4.0 (4 m), by its chemical composition and methane content, was closer to the sample taken at 2 m than to that taken at 6 m (Demidov et al. 2016). Therefore, despite the lack of a well-defined border between the Holocene and Pleistocene deposits revealed by this borehole, we presume the Holocene origin for this sample.

The borehole D-04/13 was drilled on a low sea terrace and, below the thin cover layer, penetrates the Late Pleistocene sediments. At 4 m, these sediments were radiocarbon-dated to 34300 ± 1200 years BP (Demidov et al. 2016). The sample D-04/13-3.5 collected at 3.5 m is thus not less than 30 thousand years old, assuming approximately equal sedimentation rate across the area during the Late Pleistocene. Following the same logic, the sediments of the sample D-04/13-2.5 (2.5 m) were also deposited in the Late Pleistocene.

Sediments penetrated by the borehole D-05/13 split into two benches, with a border located between 5 and 6 m. The upper bench is considered to be of the Holocene origin, while the lower formed during the Late Pleistocene (Demidov et al. 2016). Therefore, strains isolated from the samples D-05/13-2.5 and D-05/13-5.0 (2.5, 5 m, respectively) originate from the Holocene and D-05/13-6.0 (6 m) from the Late Pleistocene. The origin of sediments of the samples D-03/13-1.0 and D-07/13-2.0 cannot be determined with certainty, but the depth at which these samples were collected argues for the Holocene (Demidov et al. 2016).

**Bykovskiy Peninsula.** The Bykovskiy Peninsula harbours the most studied Late Pleistocene deposits in Siberia, called the Yedoma suite (Schirrmeister et al. 2011, Rivkina et al. 2006, Schirrmeister et al. 2002). Yedoma is formed by ice-rich loams, silts and silty sands penetrated by large ice wedges, resulting from synchronous sedimentation and freezing driven by certain climatic and environmental conditions (Schirrmeister et al. 2011, Murton et al. 2015). The boreholes D-01/01 and D-07/03 (outcrop Mamontova Hayata) yielded cores of Pleistocene loam from the very first metres, with D-07/03 penetrated into, supposedly, Pliocene sands (Rivkina et al. 2006). The sample D-01/01-2.2 (2.2 m), by the depth at which it was collected, corresponds to the unit C of Schirrmeister et al. (2002) dated 12 to 28 thousand years BP. The sample D-07/03-5.0 (5.0 m) corresponds to the unit B with the age 28–48 thousand years (Schirrmeister et al. 2002).

**Kolyma Lowland.** In this area, the Late Pleistocene Yedoma suite is also widely distributed. Samples C-02/19-1, B-34/19 and P-318-08-69a were taken from the Duvannyy Yar exposure on the Kolyma River, in its lowermost part (5–12 m above the river level). The sampled sediments were silty and sandy loams with numerous inclusions of roots and branches of shrubs which correspond to the allochthonous peat layer dated 42 to 43 thousand years (Gubin and Zanina 2013). Samples P-1084T and P-1086AT2 were taken at a similar outcrop of Stanchikovskiy Yar, located in about 100 km from Duvannyy Yar, at Malyy Anyuy River, a tributary of Kolyma. Radiocarbon dating of the sediment layer from which the samples were collected gave 34 to 37 thousand years BP (Legendre et al. 2014). A considerable part of the collection came from borehole D-03/15 drilled in the vicinity of the Allazeya riverbank, approximately in the same place as borehole 15/91 of Rivkina et al. (2006). Almost all core fragments that yielded live protists originate from the Late Pleistocene Yedoma suite, with the deepest one (14.2 m) attributed to the Olyor suite (600–1000 thousand years BP) (Rivkina et al. 2006).

## Usage rights

### Use license

Other

### IP rights notes

The GBIF dataset "Amoeboid protists isolated from ancient Siberian permafrost" (Malavin and Shmakova 2020b) is licensed under a Creative Commons Attribution Non-Commercial (CC-BY-NC) 4.0 License.

**Usage of strains from the collection**: The protist strains from the collection are freely available for non-commercial use upon request to Pushchino Scientific Center for Biological Research RAS. The distribution of strains used in the ongoing research projects will be discussed on an individual basis. The purpose of the strain usage must be stated explicitly and may be made public. Strains may not be passed to a third person without the official permission of the rights holder. The collection must be clearly referenced as the source of the strain while in public use.

## Data resources

### Data package title

Amoeboid protists isolated from ancient Siberian permafrost

### Resource link

https://www.gbif.org/dataset/e11d99cb-4a96-4e9d-847e-d078cfd59f6c

### Alternative identifiers

https://doi.org/10.15468/mfnrdv

### Number of data sets

3

### Data set 1.

#### Data set name

event.txt

#### Data format

Tab-delimited values

#### Number of columns

24

#### Character set

UTF-8

#### Description

Sampling events (i.e. boreholes, wall sampling, borrow samples) with linked occurrences (i.e. clonal cultures isolated from the samples obtained)

**Data set 1.** 

Column label	Column description
id	Sample id
dynamicProperties	Sample type in Darwin Core json format
eventID	Same as "id"
parentEventID	Parent event— usually a borehole
samplingProtocol	Sampling protocol
eventDate	Date (year) of sampling
fieldNumber	Field tag of the sample
eventRemarks	Sample description made by a collector
locationID	A link to a location of sampling at geonames.org
higherGeography	Higher geography of the sampling site
continent	Always "Asia"
country	Always "Russia"
countryCode	Country code
locality	Sampling locality
minimumDepthInMetres	The beginning depth of the core (if applicable)
maximumDepthInMetres	The ending depth of the core (if applicable)
decimalLatitude	Decimal latitude
decimalLongitude	Decimal longitude
geodeticDatum	Geodetic datum (always EPSG:4326)
georeferencedBy	Collector
georeferencedDate	Same as eventDate
earliestEpochOrLowestSeries	Earliest estimated epoch of deposit formation
latestEpochOrHighestSeries	Latest estimated epoch of deposit formation
lithostratigraphicTerms	Lithostratigraphic terms

### Data set 2.

#### Data set name

occurrence.txt

#### Data format

Tab-delimited values

#### Number of columns

24

#### Character set

UTF-8

#### Description

Characteristics of the isolated strains

**Data set 2.** 

Column label	Column description
id	Sample ID
type	Always "Collection"
language	Always "en"
rightsHolder	The rights holder, always "Pushchino Scientific Center for Biological Research RAS"
accessRights	Always "not-for-profit use only"
collectionCode	Always "SCL"
ownerInstitutionCode	Always "PSCBR"
basisOfRecord	Always"MaterialSample"
occurrenceID	Strain ID
catalogNumber	The same
disposition	"in collection" or "missing"
associatedReferences	Publications where the strain was described
associatedSequences	Publicly available sequences of the strain
organismScope	Always "clonal culture"
organismRemarks	Strain maintainance
eventID	Sample ID
eventDate	Collection date (year)
identifiedBy	By whom the strains was identified
identificationReferences	Resources used for identification
typeStatus	Type status of the strain
scientificName	Nearest possible identification following the GBIF taxonomy
scientificName	Always "Protozoa" (GBIF taxonomy)
taxonRank	Rank of the nearest identified taxon
nomenclaturalCode	Always "ICZN"

### Data set 3.

#### Data set name

extendedmeasurementsorfacts.txt

#### Data format

Tab-separated values

#### Number of columns

7

#### Character set

UTF-8

#### Description

Radiocarbon (^14^C) ages of the dated samples.

**Data set 3.** 

Column label	Column description
id	Sample ID
measurementType	Always "Age"
measurementValue	Age value
measurementAccuracy	1 standard deviation
measurementUnit	Always "years BP"
measurementMethod	Always "Radiocarbon, AMS"
measurementRemarks	Measurement remarks

## Figures and Tables

**Figure 1a.**
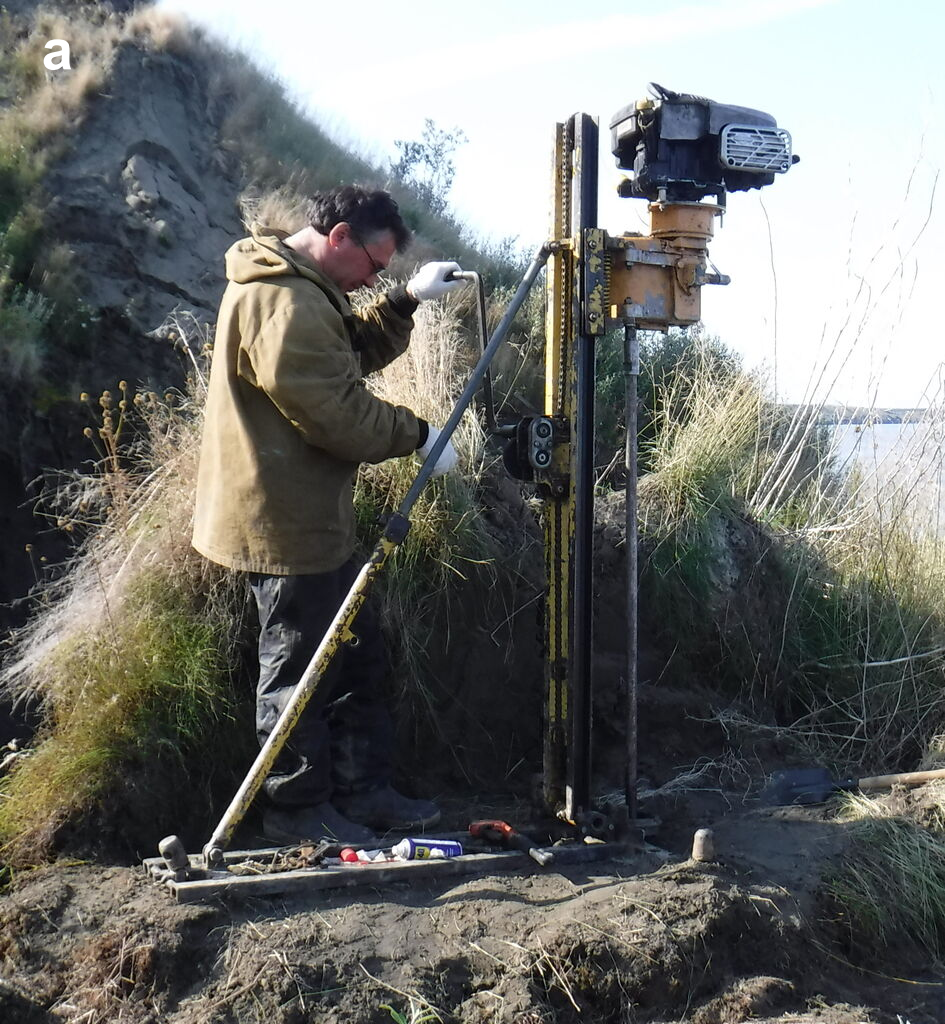
Mobile drilling rig (vertical coring).

**Figure 1b.**
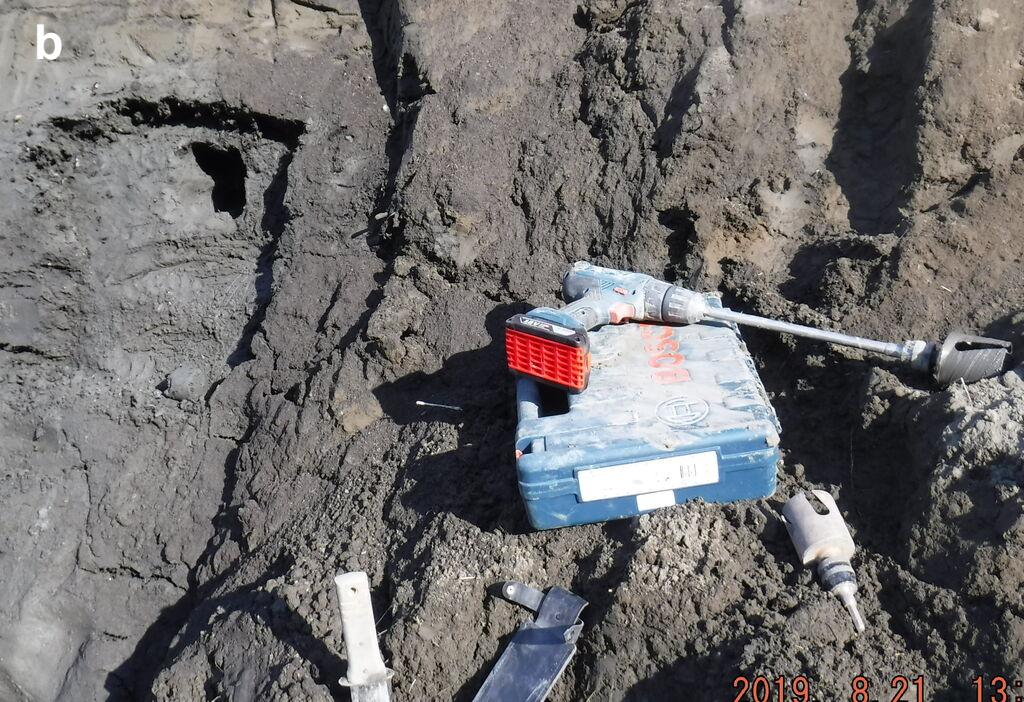
Hand drill (wall sampling).

**Figure 3a.**
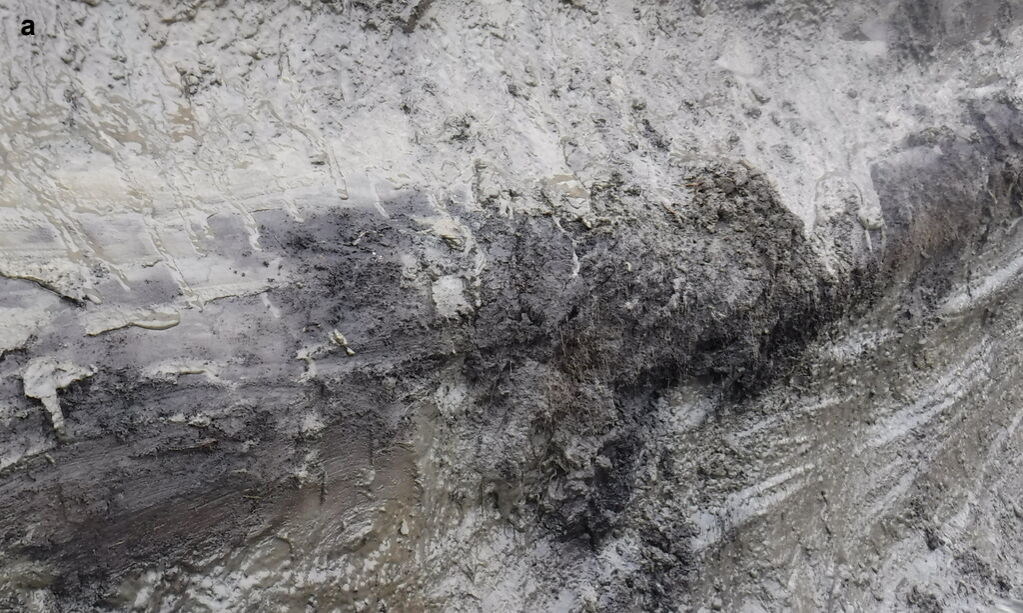
Buried Late Pleistocene soil (paleosol) at the Duvannyy Yar outcrop (Sakha Rep.).

**Figure 3b.**
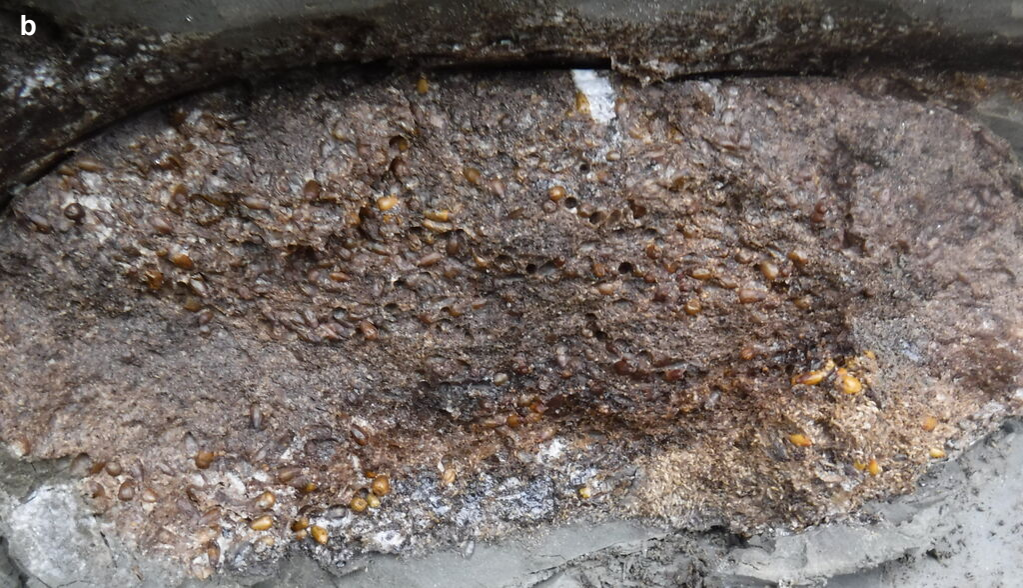
Buried terminal nesting chamber of a ground squirrel burrow. Note the supply of seeds. Duvannyy Yar.

**Table 1.** Characteristics of samples used in the study

Sampling event id	Parent event id	Year of collection	Locality	Locality link	Latitude	Longitude	Depth below surface, m
D-01/01-2.2	D-01/01	2001	Bykovskiy Peninsula	geonames.org/2025770	71.783284	129.3611761	2.16
D-07/03-5.0	D-07/03	2003	Bykovskiy Peninsula	geonames.org/2025770	71.775537	129.330297	5
D-05/13-2.5	D-05/13	2013	Ngarka-Khortiyakha River flood land, 800 m from the mouth, 100 m from the bank	geonames.org/1497773	71.463875	76.992927	2.5
D-05/13-5.0	D-05/13	2013	Ngarka-Khortiyakha River flood land, 800 m from the mouth, 100 m from the bank	geonames.org/1497773	71.463875	76.992927	5
D-05/13-6.0	D-05/13	2013	Ngarka-Khortiyakha River flood land, 800 m from the mouth, 100 m from the bank	geonames.org/1497773	71.463875	76.992927	6
D-04/13-2.5	D-04/13	2013	Southeast of the Yayne-Vonga Bay, low terrace separated from the sea by laida	geonames.org/1545199	72.348887	78.546807	2.5
D-04/13-3.5	D-04/13	2013	Southeast of the Yayne-Vonga Bay, low terrace separated from the sea by laida	geonames.org/1545199	72.348887	78.546807	3.5
D-01/13-2.0	D-01/13	2013	West of Lake Tirebyato, 10 m from the terrace cliff	geonames.org/1544900	72.350733	75.118445	2
D-01/13-4.0	D-01/13	2013	West of Lake Tirebyato, 10 m from the terrace cliff	geonames.org/1544900	72.350733	75.118445	4
D-01/13-8.0	D-01/13	2013	West of Lake Tirebyato, 10 m from the terrace cliff	geonames.org/1544900	72.350733	75.118445	8
D-03/13-1.0	D-03/13	2013	Southeast of the Yayne-Vonga Bay, laida rear welt	geonames.org/1545199	71.429709	72.991683	1
D-07/13-2.0	D-07/13	2013	Mongocheyakha River Mouth	geonames.org/1498452	N/D	N/D	2
D-03/15-3.5	D-03/15	2015	Alazeya River	geonames.org/2127297	69.3388694	154.9969472	3.5
D-03/15-14.2	D-03/15	2015	Alazeya River	geonames.org/2127297	69.3388694	154.9969472	14.2
P-1084T	P-1084	2000	Kolyma River, Stanchikovskiy Yar	geonames.org/12123736	68.370155	161.415553	N/A
P-1086AT2	P-1086	2000	Kolyma River, Stanchikovskiy Yar	geonames.org/12123736	68.370155	161.415553	N/A
P-318-08-69a	P-318-08	2008	Kolyma River, Duvannyy Yar	geonames.org/12123735	68.628232	159.194842	N/A
C-02/19-1	C-02/19	2019	Kolyma River, Duvannyy Yar	geonames.org/12123735	68.635026	159.07798	N/A
B-34/19	B-34/19	2019	Kolyma River, Duvannyy Yar	geonames.org/12123735	68.630072	159.153383	N/A

**Table 2.** Strains of the described collection. LM—Light microscopy; TEM—Transmitted electron microscopy; SEM—scanning electron microscopy; M—Molecular phylogeny; Kyr—thousands of years.

Strain	Identification	Identification basis	Sample	Location	Geological epoch	Estimated age, Kyr	Description reference	GenBank accession number (SSU)
** Amoebozoa **								
** Discosea **								
SCL-am7	Acanthamoeba sp.	LM	D-01/01-2.2	Bykovskiy Pen.	Late Pleistocene	12–28		
SCL-am8	Acanthamoeba sp.	LM, M	P-1086AT2	Kolyma Lowland	Late Pleistocene	34–37	Malavin and Shmakova 2020a	MK124583
SCL-am9	Acanthamoeba sp.	LM, M	D-07/03-5.0	Bykovskiy Pen.	Late Pleistocene	28–48		MK124584
SCL-14-2	Acanthamoeba sp.	LM, M	D-05/13-5.0	Gydan Pen.	Holocene			MK124585
SCL-14-3	Acanthamoeba sp.	LM, M	D-04/13-3.5	Gydan Pen.	Late Pleistocene	~30		MK124586
SCL-14-9	Acanthamoeba sp.	LM, M	D-05/13-6.0	Gydan Pen.	Late Pleistocene			MK124587
SCL-14-12	Acanthamoeba sp.	LM, M	P-1084T	Kolyma Lowland	Late Pleistocene	34–37		MK124588
SCL-19-2	Acanthamoeba sp.	LM	C-02/19-1	Kolyma Lowland	Late Pleistocene	42–43		
SCL-16-1	Cochliopodium sp.	LM	D-03/15-3.5	Kolyma Lowland	Late Pleistocene			
SCL-16-3	Vannella sp.	LM	D-03/15-3.5	Kolyma Lowland	Late Pleistocene			
SCL-15-5	Amoebozoa indet.	LM	D-03/15-14.2	Kolyma Lowland	Middle Pleistocene	600–1000		
SCL-14-10	Amoebozoa indet.	LM	D-05/13-2.5	Gydan Pen.	Holocene			
SCL-19-3	Amoebozoa indet.	LM	C-02/19-1	Kolyma Lowland	Late Pleistocene	42–43		
***Evosea: Variosea***								
SCL-flam1	Flamella pleistocenica Shmakova et al., 2016	LM, TEM, M	P-318-08-69a	Kolyma Lowland	Late Pleistocene	42–43	Shmakova et al. 2016	KP208180
SCL-flam2	Flamella beringiania Shmakova et al., 2016	LM, TEM, M	P-1084T	Kolyma Lowland	Late Pleistocene	34–37		KP219428
SCL-flam3	Flamella beringiania Shmakova et al., 2016	LM, TEM, M	D-04/13-3.5	Gydan Pen.	Late Pleistocene	~30		KP219429
SCL-flam4	Flamella beringiania Shmakova et al., 2016	LM, TEM, M	D-05/13-5.0	Gydan Pen.	Holocene			KP219430
SCL-flam5	Flamella pleistocenica Shmakova et al., 2016	LM, TEM, M	D-05/13-2.5	Gydan Pen.	Holocene			KP219431
SCL-flam6	Flamella beringiania Shmakova et al., 2016	LM, TEM, M	D-01/13-4.0	Gydan Pen.	Holocene			KP219432
SCL-flam9	Flamella sp.	LM	D-03/15-3.5	Kolyma Lowland	Late Pleistocene			
SCL-19-1	Flamella sp.	LM	C-02/19-1	Kolyma Lowland	Late Pleistocene	42–43		
SCL-19-8	Flamella sp.	LM	B-34/19	Kolyma Lowland	Late Pleistocene	42–43		
SCL-14-8	Filamoeba sp.	LM	D-01/13-8.0	Gydan Pen.	Late Pleistocene	15–17		
SCL-Parc	Phalansterium arcticum Shmakova et al., 2018	LM, TEM, M	D-01/13-2.0	Gydan Pen.	Holocene	8.6	Shmakova et al. 2018	KX844828
SCL-14-1	BNFA	LM	D-05/13-2.5	Gydan Pen.	Holocene			
SCL-14-4	BNFA	LM	D-05/13-6.0	Gydan Pen.	Late Pleistocene			
SCL-14-6	BNFA	LM	D-03/13-1.0	Gydan Pen.	Holocene?			
SCL-14-7	BNFA	LM	D-07/13-2.0	Gydan Pen.	Holocene?			
SCL-14-11	BNFA	LM	D-04/13-2.5	Gydan Pen.	Late Pleistocene			
SCL-16-4	BNFA	LM	D-03/15-3.5	Kolyma Lowland	Late Pleistocene			
SCL-16-5	BNFA	LM	D-03/15-3.5	Kolyma Lowland	Late Pleistocene			
** Heterolobosea **								
SCL-16-2	Heterolobosea indet.	LM	D-03/15-3.5	Kolyma Lowland	Late Pleistocene			
SCL-16-8	Heterolobosea indet.	LM	D-03/15-3.5	Kolyma Lowland	Late Pleistocene			
SCL-16-9	Heterolobosea indet.	LM	D-03/15-3.5	Kolyma Lowland	Late Pleistocene			
** Rhizaria **								
SCL-16-6	Lecythium sp.	LM	D-03/15-3.5	Kolyma Lowland	Late Pleistocene			
**Viruses**								
	Pithovirus sibericum	TEM, SEM, M	P-1084T	Kolyma Lowland	Late Pleistocene	34–37	Legendre et al. 2014	NC023423
	Mollivirus sibericum	TEM, SEM, M	P-1084T	Kolyma Lowland	Late Pleistocene	34–37	Legendre et al. 2015	NC027867
